# Screening Biophysical Sensors and Neurite Outgrowth Actuators in Human Induced-Pluripotent-Stem-Cell-Derived Neurons

**DOI:** 10.3390/cells11162470

**Published:** 2022-08-09

**Authors:** Vaibhav P. Pai, Ben G. Cooper, Michael Levin

**Affiliations:** 1 Allen Discovery Center at Tufts University, Medford, MA 02155, USA; 2 Department of Stem Cell and Regenerative Biology, Harvard University, Cambridge, MA 02138, USA

**Keywords:** bioelectricity, ion flux, membrane potential, live sensor dyes, pH, serotonin, acetylcholine, GABA, hiNSC

## Abstract

All living cells maintain a charge distribution across their cell membrane (membrane potential) by carefully controlled ion fluxes. These bioelectric signals regulate cell behavior (such as migration, proliferation, differentiation) as well as higher-level tissue and organ patterning. Thus, voltage gradients represent an important parameter for diagnostics as well as a promising target for therapeutic interventions in birth defects, injury, and cancer. However, despite much progress in cell and molecular biology, little is known about bioelectric states in human stem cells. Here, we present simple methods to simultaneously track ion dynamics, membrane voltage, cell morphology, and cell activity (pH and ROS), using fluorescent reporter dyes in living human neurons derived from induced neural stem cells (hiNSC). We developed and tested functional protocols for manipulating ion fluxes, membrane potential, and cell activity, and tracking neural responses to injury and reinnervation in vitro. Finally, using morphology sensor, we tested and quantified the ability of physiological actuators (neurotransmitters and pH) to manipulate nerve repair and reinnervation. These methods are not specific to a particular cell type and should be broadly applicable to the study of bioelectrical controls across a wide range of combinations of models and endpoints.

## 1. Introduction

Ion fluxes and membrane potential are fundamental aspects of biology and regulate cell behavior and function across species from plants and single cell organisms to vertebrates [1,2,3,4,5,6,7,8,9,10]. These ion fluxes and membrane potential mechanisms, along with biomechanical forces and canonical genetic–biochemical signals, regulate complex processes such as development, regeneration, wound healing, and disease states such as cancer [1,10,11,12,13,14,15,16,17,18,19,20,21]. The development of bioelectric interventions for regenerative medicine [22,23,24] requires an extensive database of physiological profiles of a wide range of cells, as well as methods for the study of the effects of bioelectric actuation on cell behavior. This especially concerns stem cells such as iPSCs and their derived tissues. However, few data on this are available, especially in human cells. Thus, we sought to develop methods for reading and writing dynamic bioelectric states in cell culture. Here, we report the results of testing a variety of reagents and protocols, resulting in simplified, robust methods for the use of live sensors simultaneously allowing the characterization of cell morphology, ion dynamics, membrane potential, and cell activity such as changes in pH and reactive oxygen species during nerve repair and regeneration over time. We also show the actuation of nerve repair by bioelectric modulators (neurotransmitters and pH).

Wound repair is a highly complex set of events involving precise coordination between homeostasis, inflammation, proliferation, and remodeling [25,26]. Improper repair and healing results in numerous pathologies while perfect repair (no scarring) is seen in fetal tissues and many organisms that show full regeneration [26,27,28,29]. Thus, understanding wound repair not only holds the promise of remedying ailments such as chronic wounds but also gaining insights for triggering latent regenerative abilities of tissues. Ion fluxes [30,31,32] and membrane potential [33,34,35,36,37,38] are critical regulators of wound healing and regeneration [1,20,39,40,41,42,43,44]. Nerve regeneration in wounds is also considered important for faster and better (less scaring) [45,46,47,48,49,50] wound healing, with neurite outgrowth into the wound often used as a good measure of neuronal regeneration [45,50,51,52,53,54,55,56]. There is also significant evidence for an ion flux and membrane potential mediated effect on the innervation into wounds [54,57,58,59]. Thus, we sought to develop protocols for testing bioelectric sensors and actuators in injured cultured neural tissues. We report tracking ion fluxes and membrane potential as well as neuronal injury and subsequent neurite repair and outgrowth over time. We also test biophysical actuators (pH and neurotransmitters) for modulating the neurite outgrowth postinjury.

Human induced neural stem cells (hiNSC)-derived neurons have unique advantages over both commercially available transformed neuronal cells (such as PC12 and neuroblastoma) and primary human neural cells. The commercial neural cell lines are transformed, resulting in a lack of complete neuronal functionality and a poor differentiation leading to low predictivity and less reliable results [60,61,62]. On the other hand, primary human neural cells involve ethical limitations, require extensive resources and techniques, and are difficult to obtain on a consistent basis [63,64]. In contrast, hiNSC-derived neurons have a function and behavior similar to primary neurons and are easy to culture in large quantities on a consistent manner [65,66,67,68,69,70]. Additionally, hiNSC can be driven to represent both central and peripheral neurons making them highly versatile [65,66,70]. Hence, in this study we use these hiNSC-derived neurons.

Here, we set up a culture of hiNSC-derived neurons, in which we screen several live biophysical sensors to assess the properties of living neural cells and culture generally falling into three categories: (a) cell morphology, (b) ion fluxes and membrane potential, and (c) cell activity (as indicated by changes in pH and reactive oxygen species levels). We then use some of these sensors to track and quantify neurite outgrowth in these neuronal cultures over time. Finally, we use some actuators (neurotransmitters and changes in pH) to test their effects on neurite outgrowth over time and show that nerve repair and regeneration can be modulated. These protocols and characterization data may facilitate similar approaches in a wide range of models.

## 2. Materials and Methods

### 2.1. Cell Culture

The hiNSC were a generous gift from Dr. David Kaplan, Tufts University and cultured as per the previously established protocol [65]. Briefly, hiNSC were plated on mouse embryonic fibroblast (MEF) (obtained from ATCC, Manassas, VA, USA) feeder layers that were previously inactivated with mitomycin C (Sigma-Aldrich, St. Louis, MO, USA), in hiNSC media: knockout (KO) Dulbecco’s Modified Eagle’s Medium (DMEM) (Thermo Fisher, Waltham, MA, USA) supplemented with 20% KO xeno-free SR (Thermo Fisher, Waltham, MA, USA), 20 ng/mL recombinant bFGF (Thermo Fisher, Waltham, MA, USA), 1% Glutmax (Thermo Fisher, Waltham, MA, USA), 1% antibiotic-antimycotic (Thermo Fisher, Waltham, MA, USA), and 0.1 mM β-mercaptoethanol (Thermo Fisher, Waltham, MA, USA). Media were changed every 1–3 days. For hiNSC differentiation into neurons, hiNSC growing on inactivated MEF feeder layer were trypsinized (TrypLE Select—Thermo Fisher, Waltham, MA, USA) and resuspended in differentiation media: neurobasal (Thermo Fisher, Waltham, MA, USA) media supplemented with 2% B27 (Thermo Fisher, Waltham, MA, USA), 1% Glutmax, and 1% antibiotic-antimycotic. Using manual pipetting and vortexing, cell clumps and colonies were dissociated into a single cell suspension, passed through a 40–70 µM cell strainer and plated on a cell culture surface coated with poly-d-Lysine (Thermo Fisher, Waltham, MA, USA) and laminin (Thermo Fisher, Waltham, MA, USA) in the differentiation media. Media were changed every 2 days or as needed.

### 2.2. Live Sensor Dyes

All live sensor dye tests were done on hiNSC-derived day 10 neurons grown in 96-well culture plates coated with PDL + laminin. Each experiment was done in triplicates for each condition and the experiment was repeated at least 3 times. Cell morphology dyes: Calcein Green AM (Thermo Fisher, Waltham, MA, USA) (tested 0.5–10 µM, final 0.5 µM), and Calcein Red-Orange AM (Thermo Fisher, Waltham, MA, USA) (final 0.5 µM), NeuO (StemCell Technologies, Vancouver, BC, Canada) (tested 0.1–0.3 µM, final 0.3 µM), were mixed into the culture media, and cells were incubated in the dye for 30 min (Calcein dyes) or 60 min (NeuO) followed by a wash with media followed by fluorescence imaging: Calcein Green AM (ex/em: 494/517 nm), Calcein Red-Orange AM (ex/em: 577/590 nm) and NeuO (ex/em: 470/555 nm). Nuclear dyes: DAPI (Sigma-Aldrich, St. Louis, MO, USA) (tested 0.5–10 µg/mL, final 0.5 µg/mL), Hoechst (Thermo Fisher, Waltham, MA, USA) (tested 0.5–10 µg/mL, final 0.5 µg/mL) were mixed into the culture media and added onto the cells and incubated for 5 min then the dye was removed by 3 washes with media followed by fluorescence imaging: DAPI (ex/em: 358/461 nm) and Hoechst (ex/em: 350/470 nm). Ion flux and membrane voltage dyes: intracellular Na^+^ dye, CoroNa Green AM (Thermo Fisher, Waltham, MA, USA) (tested 0.5–12.5 µM, final 12.5 µM), intracellular K^+^ dye, Asante Potassium Green—APG2 AM (Abcam, Waltham, MA, USA) (tested 1–5 µM, final 5 µM), intracellular Cl^−^ dye MQAE (Thermo Fisher, Waltham, MA, USA) (tested 0.1−10 µM, final-none worked), intracellular Ca^2+^ dye Fluo4 AM (Thermo Fisher, Waltham, MA, USA) (tested 1–5 µM, final 5 µM), and membrane potential dye DiBAC (Thermo Fisher, Waltham, MA, USA) (tested 1–12.5 µM, final 5.5 µM) were mixed into the culture media and cells were incubated in the dye for 60 min followed by two washes with media then incubation in regular media or custom Tyrode solutions for 10 min followed by fluorescence imaging: CoroNa Green AM (ex/em: 492/516 nm), APG2 AM (ex/em: 488/546 nm), MQAE (ex/em: 350/460 nm), Fluo4 AM (ex/em: 494/516 nm), and DiBAC (ex/em: 419/516 nm). Cell activity dyes: intracellular reactive oxygen species dye Peroxy Orange 1 (PO1) (R&D Systems, Minneapolis, MN, USA) (tested 1–5 µM, final 5 µM) was mixed with media and cells were incubated in the dye for 60 min. After 30 min into the incubation period, 500 µM H_2_O_2_ was added to the media. At the end of the 60 min incubation, the cells were washed twice with media followed by fluorescent imaging (ex/em: 543/565 nm). Intracellular pH dye SNARF-5F AM (Thermo Fisher, Waltham, MA, USA) (tested 5–20 µM, final 5µM) was mixed in media (serum free) and the cells were incubated in the dye for 30 min followed by media washes and an incubation in media with varying pH (6–8) with or without protonophore compound CCCP (Hello-Bio, Princeton, NJ, USA) (5 µM) for 10 min followed by fluorescence imaging (ex: 488–530 nm, em: 580 nm and 640 nm). Due to the ratiometric nature of the dye, the ratio of intensities (640 nm/580 nm) was obtained using Fiji software for the pH analysis. All imaging was done using an EVOS M7000 System (Thermo Fisher, Waltham, MA, USA).

### 2.3. Immunostaining

Briefly, hiNSC were grown in coated 96-well culture plates, fixed in 4% paraformaldehyde, washed 3 times in 1X phosphate-buffered saline (PBS), blocked in PBS with 10% goat serum and 0.1% triton X-100 for 1 h at room temperature (RT), incubated in primary antibody diluted in blocking buffer overnight at 4 °C, the following day rinsed in PBS 4 times and incubated in the corresponding fluorescently conjugated secondary antibody in blocking buffer for 1 h at RT protected from light, and finally we counterstained the nuclei with DAPI or Hoechst for 5 min at RT. Primary antibodies used were rabbit anti-βIII-tubulin (TUJ1) 1:500 (abcam-ab18207), rabbit anti-choline acetyl transferase (ChAT) 1:500 (Thermo Fisher-50-173-3063), rabbit anti-serotonin reuptake transporter (SERT) 1:500 (abcam-ab272912), and rabbit anti-glutamate decarboxylase 67 (GAD67) 1:500 (Thermo Fisher-PA5-21397). The secondary antibody used was goat anti-rabbit Alexa 488 conjugated 1:1000 (Thermo Fisher-A-11070). Imaging was done using an EVOS M7000 System (Thermo Fisher).

### 2.4. Scratch Assay

All scratch assays were done on hiNSC-derived day 10 neurons grown in 96-well culture plates coated with PDL + laminin. Each experiment was done in triplicates for each condition and the whole experiment was repeated at least 3 times. Day 10 neurons were scratch-injured mechanically using a sterile P10 pipette tip, washed with media and incubated in fresh media, media with varying pH, or media with indicated concentrations of acetylcholine, serotonin, or GABA for 48 h followed by washing with fresh media and incubation in fresh media for the rest of the experimental duration. Cells were stained with Calcein AM dye at the mentioned time points and imaged using an EVOS M7000 System (Thermo Fisher).

### 2.5. Neurite Density Quantification

Scratch-injured neural cultures stained with Calcein Red-Orange AM were used. Three random images of each scratch injury were captured from each well of the 96-well plate using Evos microscope software. Each 96-well plate had at least 3 wells for each treatment. The entire experiment was repeated greater than 3 times—independent 96-well plates were seeded from different passages of hiNSC. Using Fiji (formerly ImageJ) software, first, a control group image was adjusted to a color threshold such that the fluorescence in the fine delicate neurite projections could be clearly detected (Figure S4). This exact same color threshold derived from the control image was then applied to all the experimental group images. All images were then binarized (Figure S4). A region of interest (ROI) was selected starting from the scratch edge encompassing the area of the scratch and excluding the scratch edge and neuronal bodies (Figure S4). The ROI position and area was maintained constant through all images of an experiment. The “integrated density” function in Fiji was used to obtain a sum of values of all pixels within the ROI. For the binarized images, the integrated density function yielded a total number of nonzero pixels. The “integrated density/area of ROI” yielded a percentage of ROI covered by neurites (neurite density). Each data point on the graph represents an independent experiment (a 96-well plate with 3 wells per condition and 3 images taken per well).

### 2.6. Statistics

Statistical analyses were performed using GraphPad Prism9. In general, a minimum of three technical replicates (three wells of a 96-well plate) were maintained per condition/treatment. Each 96-well plate constituted one independent experiment. More than three independent experiments (more than three 96-well plates each seeded from different passages of hiNSC) were used for each analysis. Data were analyzed by ANOVA (for more than two groups, with Tukey’s multiple comparison test) or as indicated with each experiment. A *p*-value less than 0.05 was considered significant. Data are represented as mean ± SD.

## 3. Results and Discussion

### 3.1. Establishing Human Induced Neural Stem Cell (hiNSC)-Derived Neuron Cultures

**Figure 1:**
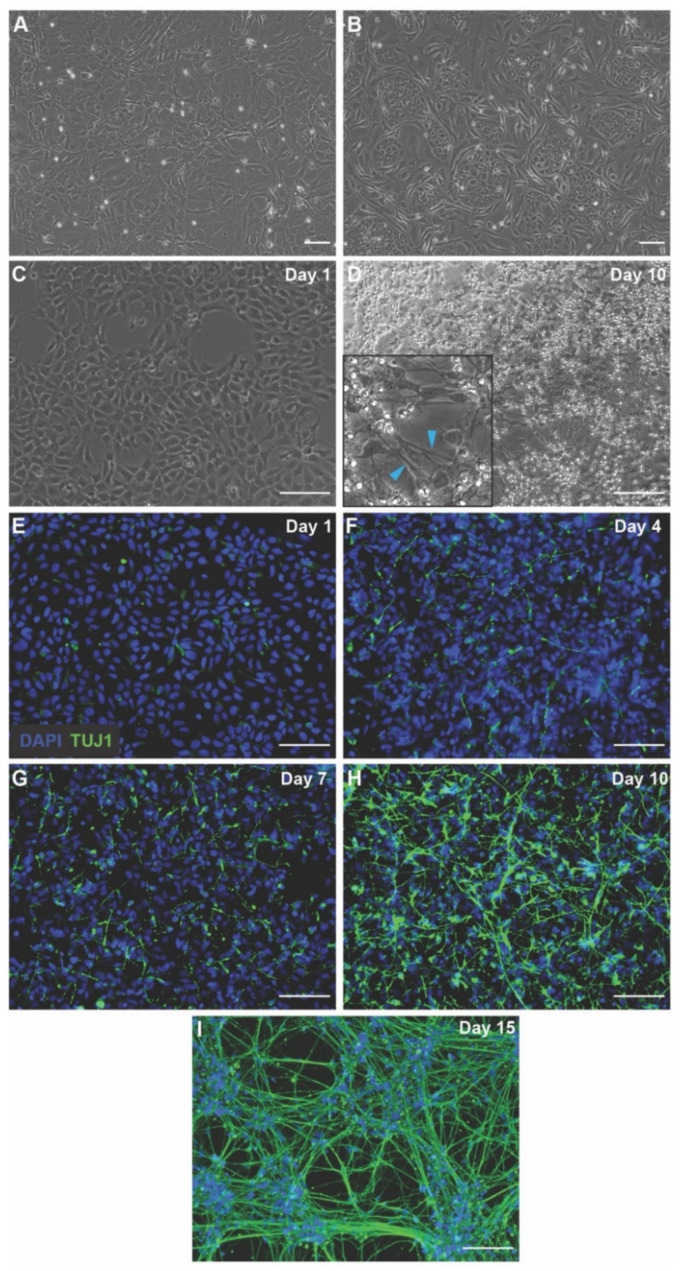
hiNSC differentiation into neurons over 10 days. (**A**) Inactivated MEFs which serve as feeder substrate for growing hiNSC. (**B**) hiNSC colonies growing on inactivated MEFs. (**C**) Day 1 after seeding hiNSC on tissue culture surface coated with poly-d-lysine (PDL) + laminin. (**D**) Day 10 after seeding hiNSC on PDL + laminin with neuronal outgrowths. Inset shows a magnified image with blue arrowheads indicating neuronal projections. (**E**) Day 1 hiNSC on PDL + laminin stained with DAPI (nucleus) and immunostained with TUJ1 (neuron-specific βIII-tubulin) showing no neurons. (**F**) Day 4 hiNSC on PDL + laminin stained with DAPI and TUJ1 showing beginning of neuronal differentiation. (**G**) Day 7 hiNSC on PDL + laminin stained with DAPI and TUJ1 showing increased progression of neuronal differentiation. (**H**) Day 10 hiNSC on PDL + laminin stained with DAPI and TUJ1 showing large-scale neuronal differentiation into neurons. (**I**) Day 10 hiNSC on PDL + laminin stained with DAPI and TUJ1 showing extensive neural networks. All scale bars, 100 µm.

Here, we build upon our previous characterization of hiNSC-derived neurons [65]. We began by characterizing hiNSC growth in vitro [65,66,67,68,69,70]. hiNSC propagated on inactivated mouse embryonic fibroblasts (MEFs) (Figure 1A) were resuspended and plated on poly-d-lysine (PDL)- and laminin-coated wells of 96-well plates with differentiation media (Figure 1B–D). Cells were fixed and stained at regular intervals (day 1, 4, 7, 10, 15) for neuron marker TUJ1 (βIII tubulin) and nuclear marker DAPI (Figure 1E,F). We observed that the TUJ1 positive neurons (seen as long green neuronal projections) started to appear slowly starting day 4 (Figure 1F). By day 10, the majority of cells stained TUJ1-positive (Figure 1H) and by day 15, they formed extensive neural networks (Figure 1I). These hiNSC-derived neural cells have previously been shown to have spontaneous calcium spiking and spontaneous action potentials [65] and here, we show a similar spontaneous calcium spiking in these cells (Movie S1 and Figure S1). Hence, we concluded that our cultures reached neuronal state by day 10, and thus used day 10 neuronal cultures for further experimentation. Since these cells can be grown in 96- or 384-well plates, they are amenable for high-throughput screens and assays which can be combined with high-content large-field-of-view image acquisition and analysis [71] to create a real-time screening protocol.

### 3.2. Live Sensors for Detecting Healthy Neurons and Their Morphology in hiNSC-Derived Neurons

The ability to assess the viability and morphology of live neurons (without affecting neuronal health) is vital in determining their health and changes in morphology under various conditions and detecting neurite outgrowth patterns during processes such as wound healing. Our dye testing results are summarized in Table 1.

**Figure 2:**
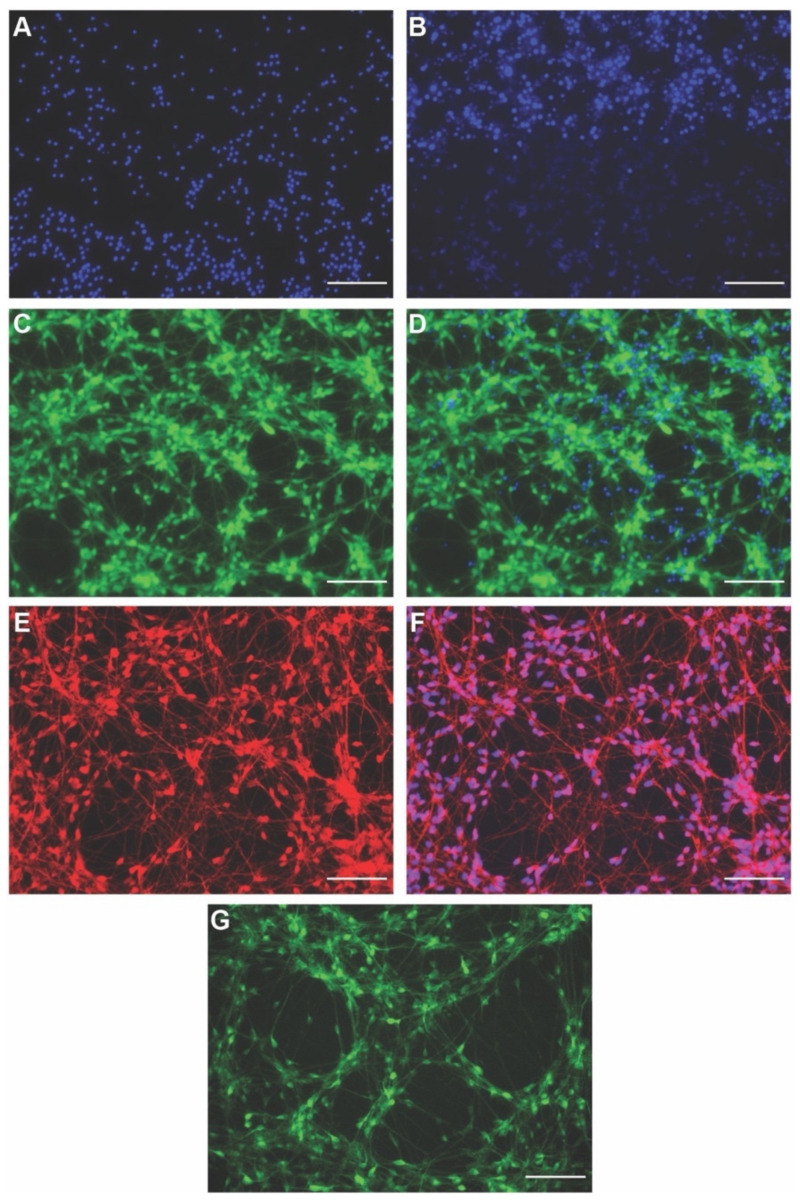
Screening live morphology dyes on hiNSC-derived neurons. (**A**–**G**) hiNSC-derived day 10 neurons. (**A**) Stained with 0.5 µg/mL DAPI showing only dead cells nuclei. (**B**) Stained with 0.5 µg/mL Hoechst showing top half (bright blue) dead cells nuclei and bottom half (light blue) live cells nuclei. (**C**) Stained with 0.5 µM Calcein Green AM showing live neurons cell body and neuronal projections. (**D**) Stained with 0.5 µM Calcein Green AM and DAPI with green showing live neuron morphology and blue showing dead cells nuclei. (**E**) Stained with 0.5 µM Calcein Red-Orange AM showing live neurons cell body and neuronal projections. (**F**) Stained with 0.5 µM Calcein Red-Orange AM and Hoechst with red/magenta showing live neuron morphology and blue showing live neuron nuclei. (**G**) Stained with 0.3 µM NeuO showing live neurons cell body and neuronal projections. All scale bars, 100 µm.

We first tested whether nuclear stains could be used while maintaining viable neurons. We tested multiple concentrations of DAPI (0.5–10 µg/mL); however, DAPI-staining heavily favored dead cells at all concentrations, with no live cell staining detectable even at 0.5 µg/mL (Figure 2A) and DAPI exposure itself was toxic to the neurons, inducing cell death. At concentrations lower than 0.5 µg/mL, DAPI stained neither dead nor live cell nuclei. We then tested Hoechst nuclear stain (0.5–5 µg/mL) [72,73]. At low concentration (0.5 µg/mL), Hoechst showed a graded staining with live neurons showing lightly stained nuclei and dead cells showing brightly stained nuclei (Figure 2B). Although Hoechst was better tolerated by neurons than DAPI, the neuron cultures still showed distress (blebbing, shorter neurites, rounded cell bodies, and loosed attachment). In addition, the dye toxicity may be further amplified by short wavelength phototoxicity which could be avoided by the use of red nuclear stains. Hence, these nuclear stains might be good sensors for counting neurons and nuclear morphology, but they are not well compatible with maintaining live cultures and are best used as endpoint assays.

To detect live neurons and see their overall morphology, we tested sensor CalceinAM-green (0.5–5 µM). A quantity of 0.5 µM CalceinAM-green was sufficient to label live neurons and beautifully show the overall morphology of neuronal cells (Figure 2C). The neurons remained healthy and brightly labeled up to 24 h postexposure (Figure 2D). Therefore, we attempted to monitor the neuron morphologies over a 24 h period with time-lapse imaging. However, we found repeated exposure to GFP wavelength (ex: 488 nm, em: 524 nm) caused significant phototoxicity in the neurons. We then attempted to monitor and visualize the neuron morphologies over multiple days by exposing them to CalceinAM-green every 24 h. However, repeated imaging and frequent exposure to CalceinAM-green also induced phototoxicity stress in the neurons under these conditions. Some of the stress could also be due to the frequent media changes and washes required for dye staining.

The frequency of exposure to CalceinAM-green that did not detrimentally affect the neurons was found to be 48 h. Considering the phototoxicity issue, we tested another version with CalceinAM-red-orange and found the red-shifted wavelengths (ex: 540 nm, em: 590 nm) to be better tolerated by the neurons without any significant phototoxicity (Figure 2E,F). Lastly, we tried NeuO, which is supposed to selectively label only neurons and indicate their viability and morphology. Among the concentrations tried (0.1–0.3 µM), 0.3 µM NeuO nicely labelled live neurons; however, the majority of the signal was mostly perinuclear and less prominent in neuronal projections, which did not indicate the morphology of the neurons very well (Figure 2G). Moreover, we found NeuO not to be selectively labelling neurons and label other non-neural cells such as macrophages (Figure S2).

Overall, we conclude that CalceinAM-red-orange serves well for determining the viability and morphology of neurons and for tracking their morphology over extended times without any detrimental impact on the health of neuronal cultures. NeuO can be used for detecting live neurons (but not their morphology) and where interest is in observing the perinuclear region of neurons.

### 3.3. Live Sensors for Detecting Changes in Intracellular Ion Concentrations and Resting Membrane Potential in hiNSC-Derived Neurons

Given the importance of bioelectrical cell states for cellular behaviors in vitro and in vivo [1,2,3,4,5,6,7,8,9,10,15,16,42,43,74,75,76,77,78,79,80,81,82,83], we next focused on testing fluorescent sensors that could report ion and voltage dynamics. We sought protocols that enabled the tracking of parameters such as changes in ion levels and membrane potential in live neurons over spatiotemporal scales in response to stimuli, injury, regeneration, or environmental change, which would not be possible with a canonical static patch clamp and voltage measurements techniques.

**Figure 3:**
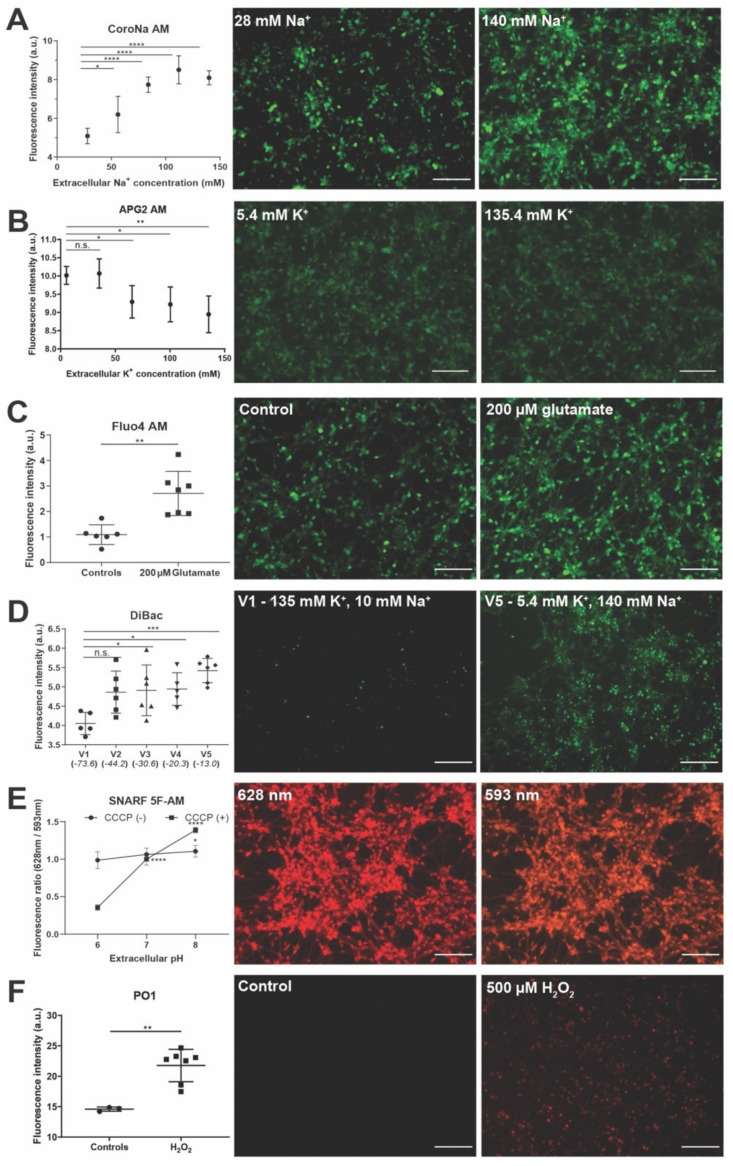
Screening live biophysical dyes showing dynamics of ions, Vmem, pH, and metabolism in hiNSC-derived neurons. (**A**–**F**) hiNSC-derived day 10 neurons. (**A**) CoroNa AM fluorescent intensity plot showing increase in intracellular Na^+^ ions in response to increasing extracellular Na^+^ ion concentrations. Representative images of CoroNa AM stained cells incubated in 28 mM and 140 mM extracellular Na^+^ ion concentration. (**B**) APG2 AM fluorescent intensity plot showing decrease in intracellular K^+^ ions in response to increasing extracellular K^+^ ion concentrations. Representative images of APG2 AM stained cells incubated in 5.4 mM and 135.4 mM extracellular K^+^ ion concentration. (**C**) Fluo4 AM fluorescent intensity plot showing increased intracellular Ca^2+^ ions in response to 200 µM glutamate. Representative images of Fluo4 AM stained cells in control and 200 µM glutamate conditions. (**D**) DiBAC fluorescent intensity plot showing increase in resting membrane voltage of cells in response to changing extracellular ion concentrations. Representative images of DiBAC stained cells in V1 (135 mM K^+^, and 10 mM Na^+^) and V5 (5.4 mM K^+^, and 140 mM Na^+^) extracellular solutions. (**E**) SNARF 5F AM fluorescence ratios (628 nm/593 nm) showing no change in intracellular pH in absence of protonophore CCCP but a significant change in intracellular pH in presence of protonophore CCCP (50 µM) in response to extracellular pH changes. Representative images of SNARF 5F AM at 628 nm and 593 nm for extracellular pH 7. (**F**) Peroxy Orange 1 (PO1) fluorescent intensity plot showing increase in intracellular reactive oxygen species (ROS) in response to hydrogen peroxide treatment. Representative images of PO1 stained cells in control and 500 µM hydrogen peroxide conditions. All data are represented as mean ± S.D. n.s.—not significant, * *p* < 0.05, ** *p* < 0.01, *** *p* < 0.001, **** *p* < 0.0001. All scale bars, 100 µm.

We first tested intracellular Na^+^ ion sensor CoroNa-AM (tested at 0.5–12.5 µM and used at 12.5 µM for all experiments). CoroNa-AM enters the cells and exhibits an increase in fluorescence emission intensity upon binding Na^+^ ions. Previously, it has been shown to detect intracellular Na^+^ in neurons within brain slices [84], and we used an established protocol for modulating extracellular Na^+^ [85]. A completely defined Tyrode solution was used as a starting extracellular solution with physiological levels of ions (high extracellular Na^+^—140 mM in comparison to cytosol). In a stepwise manner over five solutions, the Na^+^ ions were gradually replaced with impermeable *N*-Methyl-d-glucamine (NMDG^+^) with K^+^ concentration maintained at physiological levels (Table S1). This allowed us to maintain the osmolarity and electroneutrality to isolate the effect of Na^+^. Modified Tyrode solutions with Na^+^ concentration of 28 mM, 56 mM, 84 mM, 112 mM, and 140 mM (physiological level) were used as extracellular solutions to test their effect on the CoroNa-AM sensor signal. hiNSC-derived day 10 neurons were stained with CoroNa-AM followed by incubation in the modified Tyrode solutions with varying extracellular Na^+^ ions. We observed a significant (ANOVA, *p* < 0.0001) dose-dependent increase in CoroNa-AM signal (indicating an increase in intracellular Na^+^) in relation to an increase in extracellular Na^+^ (Figure 3A). Thus, CoroNa-AM successfully detects intracellular Na^+^ ion changes in live neurons and can be used to study the intracellular Na^+^ ion dynamics of neurons in response to various stimuli, injury, regeneration, and other extracellular environmental changes.

We next used Asante Potassium Green-2-AM (APG2-AM) (tested at 1–5 µM and used at 5 µM for all experiments) to monitor intracellular K^+^ ion dynamics of the hiNSC-derived neurons. APG2-AM also enters cells and exhibits an increase in fluorescence intensity upon binding K^+^ ions. To study the dynamics of K^+^ ions, we again used modified Tyrode solutions [85]. Physiologically extracellular K^+^ ions are low (5.4 mM) in comparison to cytosol. Here, over the five solutions, we slowly increased extracellular K^+^ ions with a corresponding decrease in NMDG (Table S2). Here, NMDG was used to substitute for Na^+^ to keep osmolarity constant while solely modulating K^+^. Modified Tyrode solutions with K^+^ concentrations of 5.4 mM (physiological level), 35.4 mM, 65.4 mM, 100.4 mM, and 135.4 mM were used as extracellular solutions. hiNSC-derived neurons were stained with APG2-AM followed by an incubation in the modified Tyrode solutions with varying extracellular K^+^ ions. As expected, we observed a significant (ANOVA, *p* < 0.001) dose-dependent decrease in the APG2-AM signal (indicating decreasing intracellular K^+^ ions) in relation to an increase in extracellular K^+^ (Figure 3B). Although significant (ANOVA, *p* < 0.001), the decrease in the APG2-AM signal was not as pronounced as expected. This might be because, although APG2-AM is sensitive to K^+^ ions, recently, it has been shown to also be sensitive to other cations, particularly Na^+^ [86]. Hence, the intracellular Na^+^ dynamics might still dampen the effect of APG2-AM. A newer APG4-AM sensor has been shown to have a much high sensitivity to K^+^ compared to other cations and might be better for a finer analysis of intracellular K^+^ dynamics. Nonetheless, APG2-AM successfully detects intracellular K^+^ ion changes in live neurons and can thus be used to study intracellular K^+^ ion dynamics in neurons in response to various stimuli, injury, regeneration, and other extracellular environmental changes.

Because anions are an important and highly tractable bioelectric control point [87,88], we next sought to use an MQAE sensor to detect intracellular Cl^−^ ion dynamics within hiNSC-derived neurons. MQAE has been previously used to detect intracellular Cl^−^ ion dynamics in neurons [89,90,91]. The MQAE signal intensity is inversely proportional to intracellular Cl^−^ ions. Unfortunately, after trying various concentrations of the MQAE sensor (0.1–10 mM), we were unable to detect any discernable signal above the background in the hiNSC-derived neurons (Figure S3). The majority of studies successfully employing MQAE use a two-photon microscope setup detecting MQAE via fluorescence lifetime imaging (FLIM) [89,90,91] as opposed to the conventional fluorescent microscopy used in our setup. Perhaps the use of such a FLIM imaging and analysis might allow the use of MQAE in detecting intracellular Cl^−^ dynamics in hiNSC-derived neurons. A further investigation of the use of MQAE using such a FLIM setup is warranted.

We then tested the Fluo4-AM sensor (tested at 1–5 µM and used at 5 µM for all experiments) to detect intracellular Ca^2+^ dynamics in hiNSC-derived neurons. In many cases Ca^2+^ dynamics has been used as an indirect account of changes in membrane potential of neurons [92]. Neurons showed beautiful intracellular Ca^2+^ staining as well as baseline spiking dynamics of intracellular Ca^2+^ (Figure 3C and Movie S1). However, we did notice a discernable photoactivation of intracellular Ca^2+^ dynamics in neurons while taking movies (Movie S1). This suggests that repeated exposure to the Fluo4-AM excitation wavelength (494 nm) changes the baseline intracellular Ca^2+^ dynamics, which should be carefully considered during experimentation. Glutamate is a potent inducer of free intracellular Ca^2+^ in neurons [93,94]. Incubating hiNSC-derived neurons in 200 µM glutamate for 10 min resulted in a significant (*t*-test, *p* < 0.01) and sustained increase in Fluo4-AM signal (Figure 3C) indicating a significant and sustained increase in intracellular Ca^2+^. Thus, Fluo4-AM successfully detects intracellular Ca^2+^ ion changes in live neurons and can thus be used to study intracellular Ca^2+^ ion dynamics in neurons in response to various stimuli, injury, regeneration, and other extracellular environmental changes in addition to using it as a surrogate for membrane voltage changes.

Although intracellular Ca^2+^ dynamics have been used as an indirect indicator of membrane voltage change [92,95,96,97], there is not always a direct correlation between the two as Ca^2+^ also serves as a secondary messenger for multiple cellular signaling pathways [95,98,99], while transduction pathways other than Ca^2+^ can operate downstream of voltage change [1]. Most importantly, while fast-response dyes such as calcium dyes are ideal for characterizing spiking, the slowly changing resting potential states critical for the control of proliferation and differentiation are best visualized with slow-response dyes. Here we use the DiBAC [100,101] voltage reporter dye (tested at 1–12.5 µM and used at 5.5 µM for all experiments) for a direct measurement of the resting membrane potential of hiNSC-derived neurons. As the cells depolarize (increase in resting membrane potential), DiBAC fluorescence increases [102]. To induce changes in the resting membrane potential of neurons, we again used modified Tyrode solutions for extracellular conditions [85]. Using five solutions, we gradually increased K^+^ and decreased Na^+^ (Table S3). This strategy has been shown to progressively increase the resting membrane potential of cells [85]. Using the Goldman–Hodgkin–Katz equation, the membrane potential for the five solutions were calculated (−73.6 mV, −44.2 mV, −30.6 mV, −20.3 mV, and −13 mV). hiNSC-derived neurons were stained with DiBAC followed by incubation in the modified Tyrode solutions for 10 min. As expected, we observed a significant increase in the DiBAC signal (suggesting depolarization) in relation to depolarizing extracellular solutions (Figure 3D). Thus, DiBAC successfully detects resting membrane potential changes in live neurons and can be used to study resting potential dynamics of neurons in response to various stimuli, injury, regeneration, and other extracellular environmental changes. Furthermore, the resting membrane potential changes can be visualized in combination with intracellular Ca^2+^.

Thus, overall ion and membrane voltage sensors can be used for monitoring bioelectric parameters in live neurons. These sensors can also be used independently or in combination; for example, the membrane voltage and intracellular Ca^2+^ can be monitored simultaneously to tease apart the independent and/or overlapping roles of the two during any event [98]. In addition, it can be concluded that the modified Tyrode solutions can be used as actuators to bring about a particular change in the bioelectric parameter to investigate the role of specific ions or membrane potential in a cellular phenomenon.

### 3.4. Live Sensors for Detecting Changes in Cellular State Such as pH and Metabolism in hiNSC-Derived Neurons

Intracellular pH is tightly linked to the excitability of neurons due to its effect on ion channels [103,104]. An electrical activity can lead to rapid changes in pH making the mechanisms that regulate pH very important. As a result, intracellular pH is an important determinant of both physiological and pathophysiological conditions of neurons [103,104]. Moreover, environments such as wounds have a very dynamic pH, which in turn might affect intracellular pH [105,106,107,108]. Hence, monitoring intracellular pH in neurons is important to characterize neuronal health and function. To monitor the intracellular pH of hiNSC-derived neurons, we used the SNARF-5F-AM sensor (tested at 5−20 µM and used at 5 µM for all experiments). SNARF-5F-AM allows the ratiometric measurement of fluorescence at two wavelengths (640 nm/580 nm) allowing the quantitative determination of intracellular pH. hiNSC-derived neurons stained with SNARF-5F-AM showed a nice fluorescent signal at both wavelengths. To test the sensor, we prepared media with three different pH values (pH 6, 7, and 8). After incubating the neurons in media with different pH values for 10 min, we saw a small but significant increase in ratio from pH 6–8 (Figure 3E). This observation is particularly interesting because generally, cells are impermeable to protons (H^+^) and thus largely shield their intracellular pH from extracellular proton changes. Hence, it appears that the SNARF-5F-AM sensor is sensitive enough to detect even minor changes in intracellular pH. To further test the sensitivity of SNARF-5F-AM, we used a protonophore CCCP (5 µM) along with media ranging in pH between 6 and 8. This would allow the transfer of protons between extracellular and intracellular solutions, thus forcing a change in the intracellular pH of neurons. Under these conditions we observed a large and significant increase in fluorescence ratio from pH 6–8 (Figure 3E). Thus, SNARF-5F-AM successfully detects changes in intracellular pH in live neurons and can be used to monitor the intracellular pH of neurons while testing various conditions of stimuli, injury, regeneration, and other environmental changes. Changing extracellular pH can be used as a modality for changing the intracellular pH of neurons albeit to a very small extent.

Neurons are metabolically active cells and reactive oxygen species (ROS) are largely byproducts of cellular metabolism [109,110,111]. Baseline levels of ROS are important in neurons for regulating the signaling involved in differentiation, polarization, and synaptic plasticity, while excessive ROS accumulation results in oxidative damage to neurons [109,110,111]. Hence, monitoring intracellular ROS in neurons is important to determine neuronal health. To monitor intracellular reactive oxygen species (ROS) in hiNSC-derived neurons, we used a Peroxy Orange 1 (PO1) sensor (tested at 1–5 µM and used at 5 µM for all experiments) [112]. Once inside the cell, PO1 responds to ROS and emits a fluorescence with an intensity proportional to intracellular ROS. To test the sensor, we used media with 500 µM hydrogen peroxide (H_2_O_2_). After incubating the neurons in the media with H_2_O_2_ for 10 min, we observed a significant increase in PO1 signal (Figure 3F). Thus, PO1 successfully detects changes in intracellular ROS in live neurons and can be used to monitor the metabolic health of neurons under various conditions of stimuli, injury, regeneration and other environmental changes.

Overall, the pH and ROS sensors can be used to monitor the cell state in live neurons. These sensors can be used in any combination with the morphology dyes and the bioelectric sensors to monitor neuronal dynamics based on specific experimental variables. Altogether, the panel of morphology, bioelectric, and cell state sensors characterized above serve as powerful tools for monitoring intracellular biophysical dynamics in live neurons.

### 3.5. Establishing Scratch Assay for Neuronal Regeneration and Quantitative Determination of Neurite Outgrowth from Injured Neurons Using hiNSC-Derived Neurons

**Figure 4:**
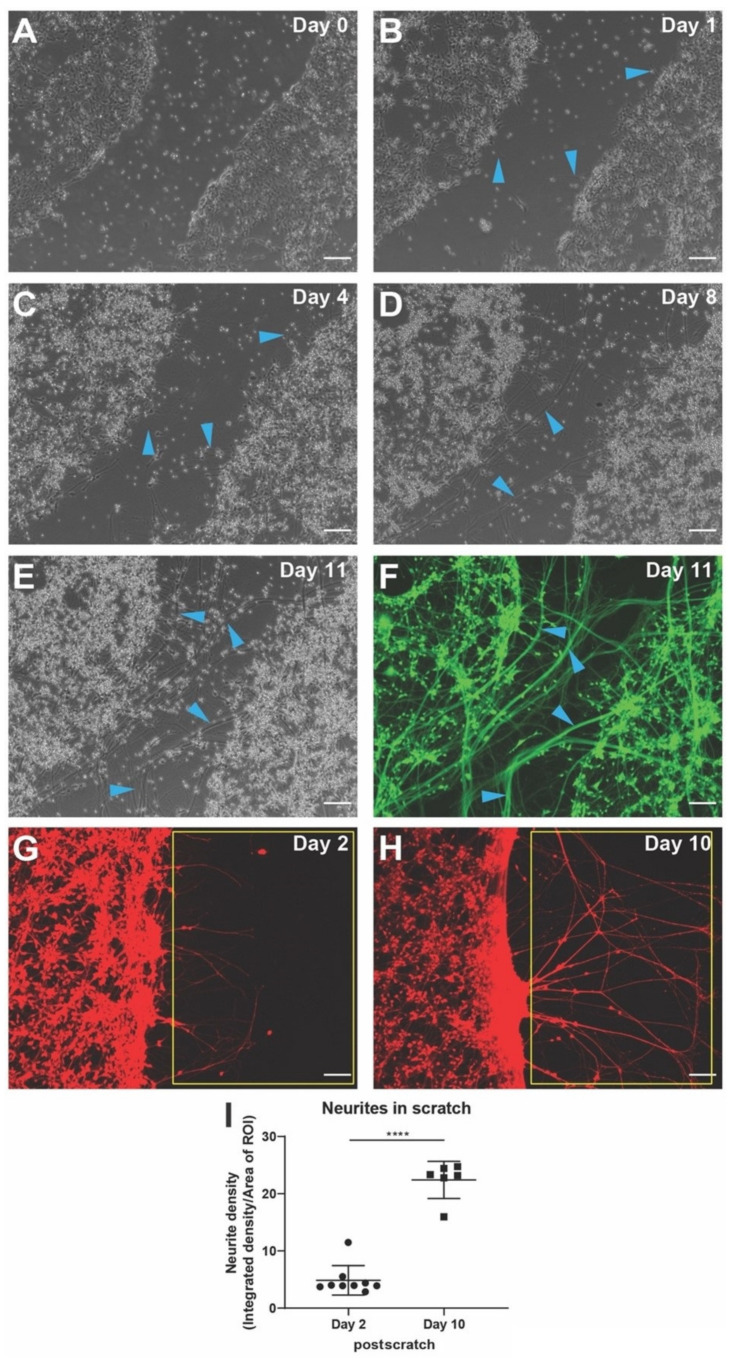
Scratch assay for neuronal injury and quantitative determination of neurite outgrowth from injured neurons. (**A**) hiNSC-derived day 10 neurons with a scratch injury. (**B**) Day 1 post-scratch, sparse rudimentary outgrowth by some cells into the scratch are seen (blue arrowheads). (**C**) Day 4 post-scratch shows small, localized network of outgrowths between close neighboring cells within the scratch (blue arrowheads). (**D**) Day 8 post-scratch, large thick neural networks and neural fibers are seen throughout the scratch (blue arrowheads). (**E**) Day 11 post-scratch, the entire scratch is covered with neural network and thick neural bundles can be observed traversing across the scratch (blue arrowheads). (**F**) Calcein Green AM live stain on day 11 post-scratch shows live neurons with extensive neurite outgrowth and nerve fibers throughout the scratch. (**G**–**H**) Calcein Red-Orange AM live staining of day 2 post-scratch (**G**) and day 10 post-scratch (**H**) for quantifying overall neurite outgrowth. Yellow box indicates the region of interest for measuring neurite outgrowth as intensity density. (**I**) hiNSC-derived neurons show a significant increase in neurite density between day 2 and day 10 post-scratch. All data are represented as mean ± S.D. **** *p* < 0.0001. All scale bars, 100 µm.

Wounding involves nerve injury in addition to many other aspects, and neuronal regeneration and neurite outgrowth is an important determinant of proper wound healing [45,50,51,52,53,54,113]. Scratch injury assays are routinely performed to study injury and subsequent recovery [56]; thus, we sought to establish methods to characterize bioelectric states during this process. Confluent hiNSC differentiated into neurons over ten days were subjected to mechanical scratching (Figure 4A). The scratch location was monitored and imaged till day 11 post-scratch (Figure 4A–F). On day 1 post-scratch, sparse small projections were given out into the scratch by neurons lining the scratch (Figure 4B). By day 4 post-scratch, we observed that the small projections had grown into small, localized network of neurite outgrowths in the scratch connecting neighboring neurons (Figure 4C). By day 8 post-scratch, they had grown into large neurite networks within the scratch with thick neurite fibers traversing across the scratch injury (Figure 4D). By day 11 post-scratch, it had grown into an extensive dense network of neurite outgrowth and thick neurite fibers covering the entire scratch injury (Figure 4E). The live morphology sensor Calcein Green AM showed that these neurons, neurite networks, and fibers were alive and healthy (Figure 4E). Given the technical problems with multiday repeated use of Calcein Green AM as discussed above, we used the well-tolerated Calcein Red-Orange AM over ten days post-scratch to track and quantify neurite outgrowth (Figure 4G–I). We observed a marked increase in neurite density within the scratch injury between day 2 and day 10 post-scratch. This scratch injury assay and neurite density quantification was used to test the effect of various actuators/stimuli on neuronal regeneration and recovery postinjury.

### 3.6. Acetylcholine Has Biphasic Effect on Neurite Outgrowth after Scratch Injury

Neurotransmitter signaling is both a canonical transduction step downstream, and a trigger upstream, of voltage dynamics, not only in neurons but also in non-neural cells [114,115,116,117]. Much work has gone into serotonergic endpoints in bioelectric signaling [118,119,120], but the effects of neurotransmitters on the repair by hiNSC derivatives are largely unknown. Thus, we used our system to examine functional impacts of a number of neurotransmitters, starting with acetylcholine, which is known to affect the central and peripheral nervous system as well as non-neural tissues through its ionotropic (direct influence on membrane voltage) and metabotropic (complex long-term effects) receptors [121,122].

**Figure 5:**
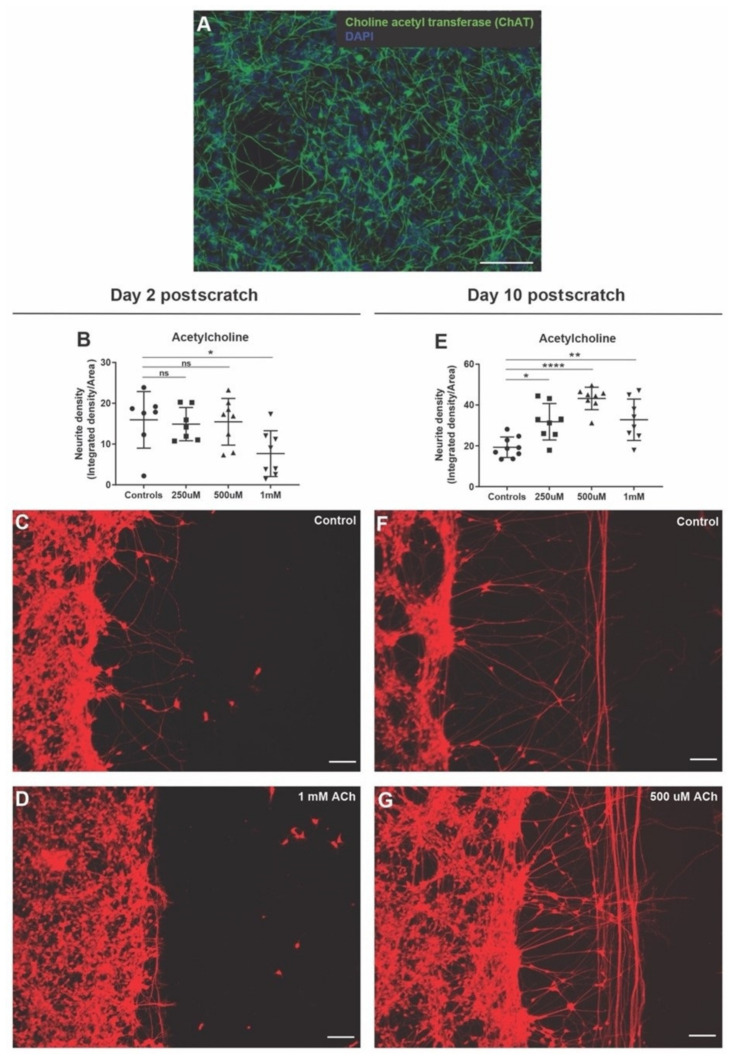
Acetylcholine shows biphasic effect on neurite outgrowth in scratch assay. (**A**) hiNSC-derived day 10 mature neuronal culture shows presence of cholinergic neurons (choline acetyl transferase marker). (**B**) Acetylcholine treatment (48 h) shows a significant concentration-dependent decline in scratch neurite density on day 2 post-scratch. (**C**,**D**) Representative images of Calcein Red-Orange AM stained control neural cultures (**C**) and neural cultures treated with 1 mM acetylcholine (**D**) on day 2 post-scratch showing diminished neurite outgrowths with acetylcholine treatment. (**E**) Acetylcholine treatment (48 h) shows a significant concentration-dependent increase in scratch neurite density on day 10 post-scratch. (**F**,**G**) Representative images of Calcein Red-Orange AM stained control neural cultures (**F**) and neural cultures treated with 500 µM acetylcholine (**G**) on day 10 post-scratch showing increased neurite outgrowth with acetylcholine treatment. All data are represented as mean ± S.D. ns—not significant, * *p* < 0.05, ** *p* < 0.01, **** *p* < 0.0001. All scale bars, 100 µm.

To test the effect of acetylcholine on neurite outgrowths after injury, we first checked if hiNSC-derived neurons had cholinergic neurons. Immunostaining for choline acetyl transferase showed cholinergic neurons distributed evenly throughout the cultures (Figure 5A). Neurons were then mechanically injured as mentioned above and left untreated (controls) or treated with three different concentrations of acetylcholine (250 µM, 500 µM, 1 mM) for 2 days. On day 2 post-scratch, the 250 µM and 500 µM acetylcholine-treated scratch injuries were indistinguishable from the controls (Figure 5B). However, 1 mM acetylcholine significantly reduced the number of neurite outgrowth and neurite density (Figure 5B–D). Contrarily, day 10 post-scratch showed a significant increase (in comparison to the controls) in overall neurite density in the acetylcholine treatment group, which peaked at 500 µM and then fell back a bit at 1 mM (Figure 5E–G). Although the increase in neurite density was striking (Figure 5F,G), we observed this increase mainly in the number of neurite fibers running parallel to the scratch edge. The neurite outgrowths away from the scratch surface (perpendicular to the scratch) were the same if not reduced in comparison to the controls (Figure 5F,G). Thus, acetylcholine seems to show a biphasic effect on neurite outgrowth with a suppression of neurite outgrowth on day 2 post-scratch but an enhanced neurite density by day 10 post-scratch with neurite fibers mainly arranged parallel to the scratch, a sign of impaired nerve repair and regeneration.

### 3.7. Serotonin Significantly Enhances Neurite Outgrowth after Scratch Injury

**Figure 6:**
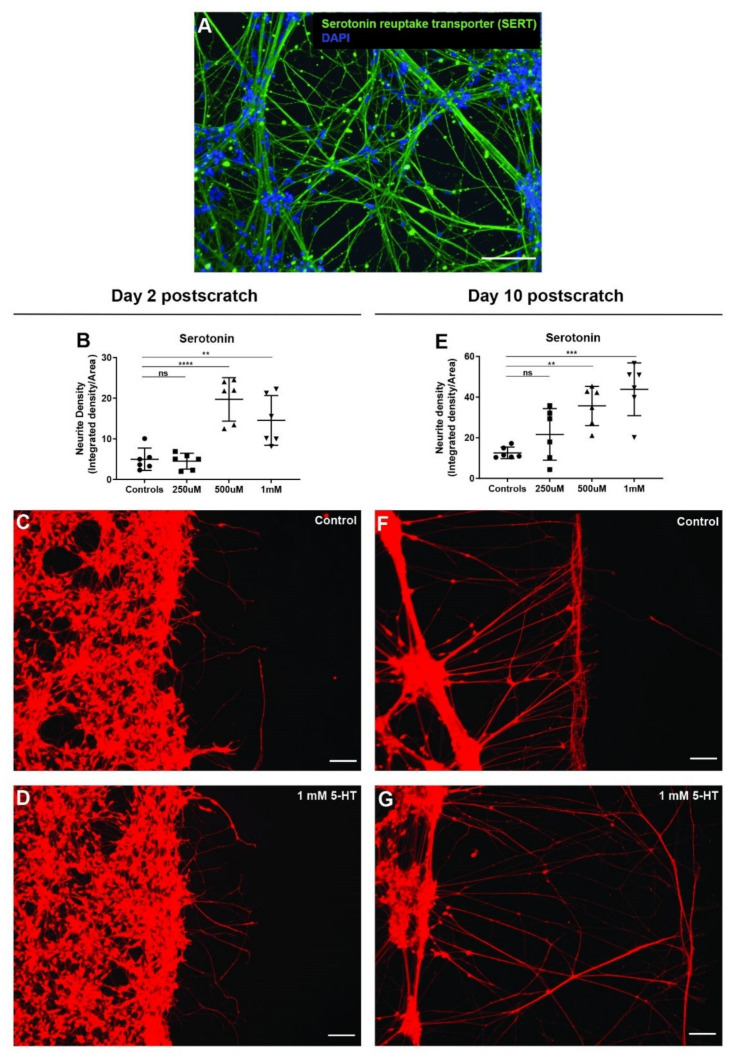
Serotonin significantly increases neurite outgrowth in scratch assay. (**A**) hiNSC-derived day 10 neuronal culture shows presence of serotonergic neurons (serotonin reuptake transporter marker). (**B**) Serotonin treatment (48 h) shows a significant concentration-dependent increase in scratch neurite density on day 2 post-scratch. (**C**,**D**) Representative images of Calcein Red-Orange AM stained control neural cultures (**C**) and neural cultures treated with 1 mM serotonin (**D**) on day 2 post-scratch showing increased neurite outgrowths with serotonin treatment. (**E**) Serotonin treatment (48 h) shows a significant concentration-dependent increase in scratch neurite density on day 10 post-scratch. (**F**,**G**) Representative images of Calcein Red-Orange AM stained control neural cultures (**F**) and neural cultures treated with 1 mM serotonin (**G**) on day 10 post-scratch showing increased neurite outgrowth with serotonin treatment. All data are represented as mean ± S.D. ns—not significant, ** *p* < 0.01, *** *p* < 0.001, **** *p* < 0.0001. All scale bars, 100 µm.

To test the effect of serotonin on neurite outgrowths after injury, we first checked if hiNSC-derived neurons had serotonergic neurons. Immunostaining for serotonin reuptake transporter showed bundles of serotonergic neurons within the cultures (Figure 6A). Neurons were then mechanically injured as mentioned above and left untreated (controls) or treated with three different concentrations of serotonin (250 µM, 500 µM, 1 mM) for 2 days. On day 2 post-scratch, the 250 µM serotonin-treated scratch injuries were indistinguishable from controls (Figure 6B). However, 500 µM and 1 mM serotonin significantly increased the number of neurite outgrowth and neurite density (Figure 6B–D). Analogously, day 10 post-scratch showed a significant dose-dependent increase (in comparison to the controls) in overall neurite density in the serotonin treatment group (Figure 6E,G). The increase in neurite outgrowth was very striking (Figure 6F,G), with much longer neurite outgrowths (perpendicular to the scratch surface) and very few fibers parallel to the scratch surface. Such extensive neurite outgrowth pattern is known to be conducive to nerve regeneration and wound healing [45,50,51,52,53,54,113,123]. Thus, serotonin exposure significantly enhances neurite outgrowths and serotonin treatment may aid in nerve repair and regeneration postinjury.

### 3.8. GABA Has No Effect on Neurite Outgrowth after Scratch

**Figure 7:**
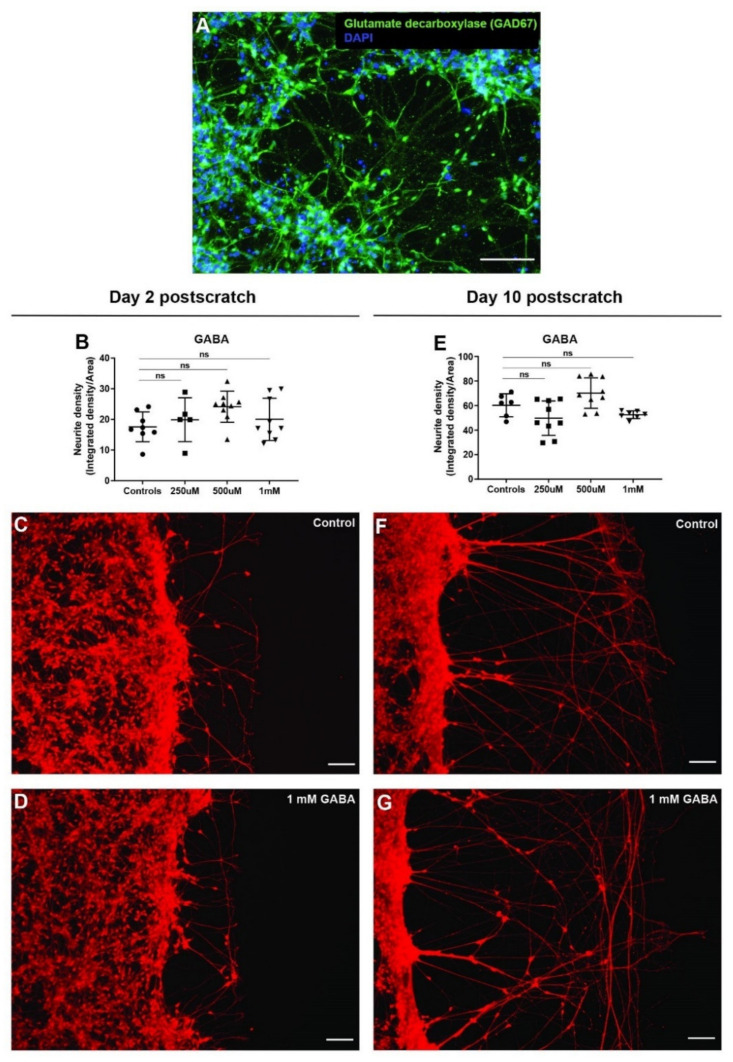
GABA does not affect neurite outgrowth in scratch assay. (**A**) hiNSC-derived day 10 neuronal culture shows presence of GABAergic neurons (glutamate decarboxylase—GAD67 marker). (**B**) GABA treatment (48 h) shows no significant change in scratch neurite density on day 2 post-scratch. (**C**,**D**) Representative images of Calcein Red-Orange AM stained control neural cultures (**C**) and neural cultures treated with 1 mM GABA (**D**) on day 2 post-scratch showing no discernable change in neurite outgrowths with GABA treatment. (**E**) GABA treatment (48 h) shows no significant change in scratch neurite density on day 10 post-scratch. (**F**,**G**) Representative images of Calcein Red-Orange AM stained control neural cultures (**F**) and neural cultures treated with 1 mM GABA (**G**) on day 10 post-scratch showing no discernable change in neurite outgrowth with GABA treatment. All data are represented as mean ± S.D. ns—not significant. All scale bars, 100 µm.

To test the effect of GABA on neurite outgrowths after injury, we first checked if hiNSC-derived neurons had GABAergic neurons. Immunostaining for glutamate decarboxylase showed GABAergic neurons within the cultures (Figure 7A). Neurons were then mechanically injured as mentioned above and left untreated (controls) or treated with three different concentrations of GABA (250 µM, 500 µM, 1 mM) for 2 days. Both on day 2 post-scratch and day 10 post-scratch, there was no discernable effect of GABA at any of the concentrations tested (Figure 7B–G). The neurite outgrowth density and patterns were indistinguishable from the controls. Thus, although GABAergic neurons are present in culture, targeting them using GABA does not affect the neurite outgrowths and hence nerve repair and regeneration. However, the loss of function (blockade) of native GABA signaling could be a target in future studies.

### 3.9. Extracellular pH Change Has Biphasic Effect on Neurite Outgrowth after Scratch Injury

**Figure 8:**
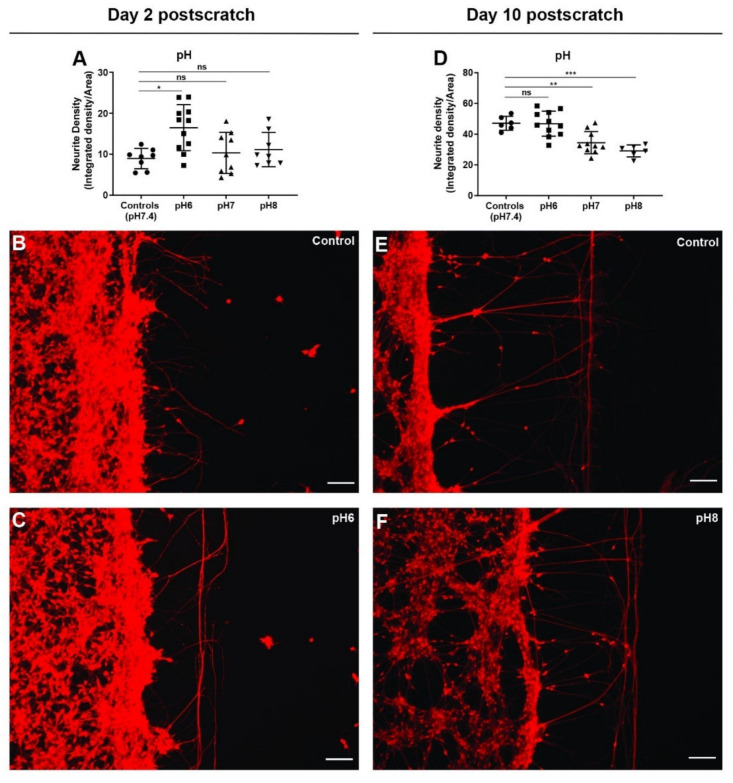
Extracellular pH change has biphasic effect on neurite outgrowth in scratch assay. (**A**) Extracellular pH change (48 h) shows a significant increase in scratch neurite density at pH 6 but no change at pH 8 on day 2 post-scratch. (**B**,**C**) Representative images of Calcein Red-Orange AM stained control neural cultures (**B**) and neural cultures with extracellular pH 6 (**C**) on day 2 post-scratch showing increased neurite outgrowths with pH 6. (**D**) Extracellular pH change (48 h) shows a significant decrease in scratch neurite density at both pH 7 and pH 8 but no change in pH 6 on day 10 post-scratch. (**E**,**F**) Representative images of Calcein Red-Orange AM stained control neural cultures (**E**) and neural cultures treated with extracellular pH 8 (**F**) on day 10 post-scratch showing decreased neurite outgrowth with pH 8. All data are represented as mean ± S.D. ns—not significant, * *p* < 0.05, ** *p* < 0.01, *** *p* < 0.001. All scale bars, 100 µm.

Wound environments in general have elevated pH which is detrimental to wound healing, and acidic pH has been shown to promote wound healing by various means such as altering protease activity, enhancing epithelialization, and angiogenesis [105,106,107,108]. To test the effect of pH on nerve repair and regeneration, hiNSC-derived neurons were mechanically injured as mentioned above and left untreated (controls) or incubated in regular culture media with pH adjusted to 6, 7, and 8 for 2 days. On day 2 post-scratch, neurons in pH 7 and pH 8 media had scratch injuries indistinguishable from those of the controls (pH 7.4) (Figure 8A). However, neurons in acidic pH 6 media showed a significantly higher neurite density (Figure 8A–C). However, the increase in neurite outgrowth pattern was mainly in the form of increased fibers parallel to the scratch surface, which is characteristic of impaired nerve repair and regeneration (Figure 8C). In contrast, on day 10 post-scratch, neurons exposed to pH 6 media were not different from the controls (pH 7.4); however, neurons exposed to both pH 7 and pH 8 showed a significant decrease in neurite density and outgrowth (Figure 8D–F). Thus, overall, it seems that acidic pH is better for neurite outgrowth and an elevated pH impairs nerve repair and regeneration. This is in line with studies [105,106,107,108] showing that an acidic wound environment promotes better wound healing. Interestingly, the controls at a physiological pH of 7.4 seemed to violate this trend at day 10, which may be due to an absence of pH shock on day 2 (going from pH 7.4 to pH 7.4 as opposed to going from pH 6 to pH 7.4) or a homeostatic sweet spot at a physiological pH of 7.4. Nonetheless, the modulation of pH could be used as a strategy to boost nerve repair and regeneration post-injury.

Here, we characterized a number of reagents that allow the dynamic monitoring of cell morphology, membrane voltage, ion levels, and cell activity such as pH and reactive oxygen species, tracked responses to injury and reinnervation, and showed in a quantitative manner the ability of bioelectrical actuators (neurotransmitters and pH) to manipulate nerve repair and regeneration. Although our neurite scratch density measurements were very successful in detecting and quantifying changes in neurite outgrowths, they did not seem to capture the differences in patterns of neurite outgrowth. Hence, additional quantitative measures such as computing the neurite length and direction might have to be added for capturing the changes in the patterns of neurite outgrowths [123,124]. Ultimately, these hiNSC-derived neurons can be cocultured with glia, astrocytes, oligodendrocytes or with muscles, skin cells, etc., [65,66] to further study the bioelectric and neurotransmitter signaling during the complex interactions of these cells under various stimuli and experimental conditions. Future work on the biophysics of cultured stem cells will continue to enrich efforts to decipher the workings of cellular collectives during wound healing, regeneration, and other environmental challenges, with many biomedical applications.

## Figures and Tables

**Table 1:** List of biophysical dyes and their outcome with hiNSC derived neurons.

	Sensor Dye	Purpose	Live/Endpoint	Works/Does Not Work
Morphology	DAPI	Nuclear stain	Endpoint	√
	Hoechst	Nuclear stain	Endpoint	√
	NeuO	Cell morphology	Live stain	✕
	Calcein AM—Green	Cell morpholgy	Live stain	√
	Calcein AM—Red	Cell morphology	Live stain	√
Vmem & ion flux	DiBac	Membrane voltage	Live stain	√
	CoroNA AM	Intracellular Na^+^	Live stain	√
	APG-2 AM	Intracellular K^+^	Live stain	√
	Fluo4 AM	Intracellular Ca^2+^	Live stain	√
	MQAE	Intracellular Cl^−^	Live stain	✕
Cell activity	SNARF-5F AM	Intracellular pH	Live stain	√
	PO1	Intracellular ROS	Live stain	√

## Data Availability

All data generated or analyzed during this study are included in this article and its Supplementary Materials and are available from the corresponding author upon request.

## Supplementary Materials

The following are available online at https://www.mdpi.com/article/10.3390/cells11162470/s1, Figure S1: Calcium spiking behavior in hiNSC-derived neurons, Figure S2: NeuO stains non-neural M0 macrophage cells, Figure S3: MQAE live stain fails to label hiNSC derived neurons, Figure S4: Quantitative determination of neurite outgrowth from scratch-injured neurons, Movie S1: hiNSC-derived day 10 neurons stained with Fluo4 AM showing baseline Ca^2+^ dynamics in neurons, Table S1: Ionic composition (in mM) of extracellular solutions with changing Na^+^ ion concentration and physiological levels in green, Table S2: Ionic composition (in mM) of extracellular solutions with changing K^+^ ion concentration and physiological levels in green, Table S3: Ionic composition (in mM) of extracellular solutions within changing Na^+^ and K^+^ ion concentration for generating various resting membrane potentials with physiological levels in green.
