# Supplementary material for: Screening Biophysical Sensors and Neurite Outgrowth Actuators in Human Induced-Pluripotent-Stem-Cell-Derived Neurons
Source: Cells. 2022 Aug 9;11(16):2470. doi: 10.3390/cells11162470 (PMC9406775; doi:10.3390/cells11162470)
Supplement: Supplementary file 1 [file cells-11-02470-s001.zip › cells-1825736-supplementary materials.pdf]

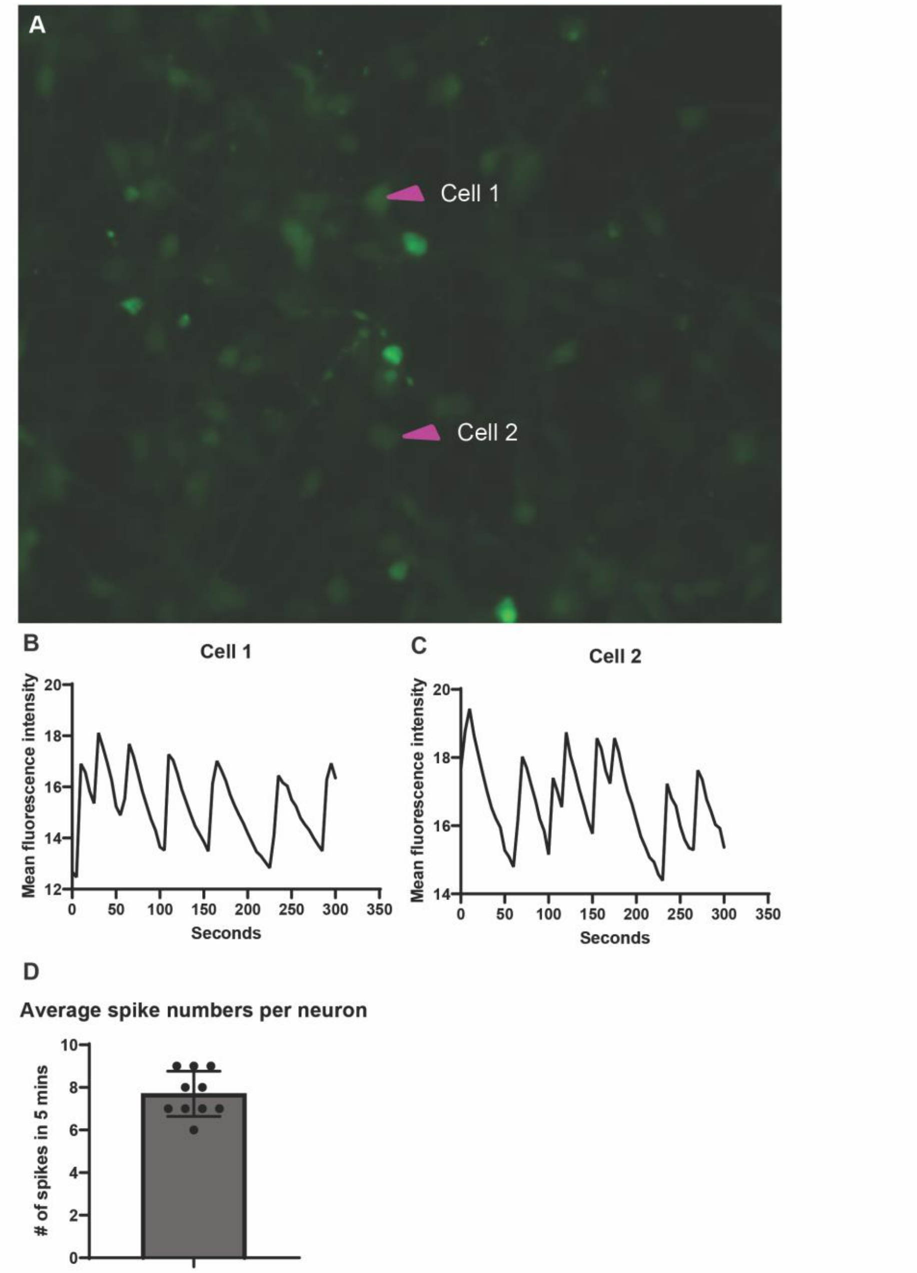

Figure S1. Calcium spiking behavior in hiNSC derived neurons. hiNSC derived day 10 neurons stained with Fluo4 AM an intracellular Ca^{2+} ion reporter and imaged for 5 min at 5 s interval as depicted in movie S1. (A) first frame of the movie indicating the two cells whose fluorescence was quantified. (B-C) Quantification of changes in fluorescent signal in two indicated cells over 5mins showing calcium spiking in the cells. (D) Average number of calcium spikes in neurons over 5 min.

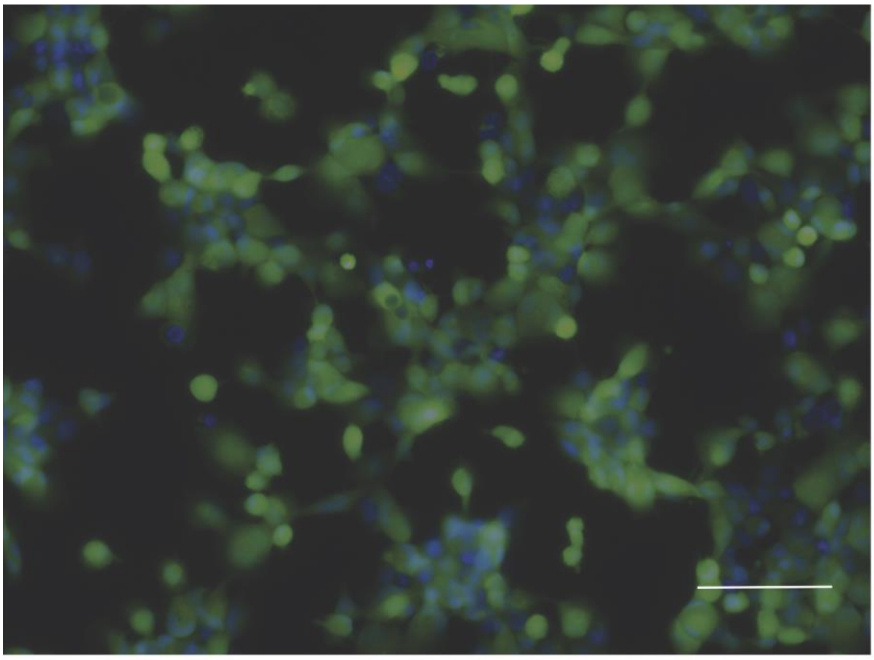

Figure S2. NeuO stains non-neural M0 macrophage cells. THP-1 derived M0 macrophage cells stained with NeuO (green) showing live cell body and Hoechst (blue) showing nuclei. Scale bars, 100 μm.

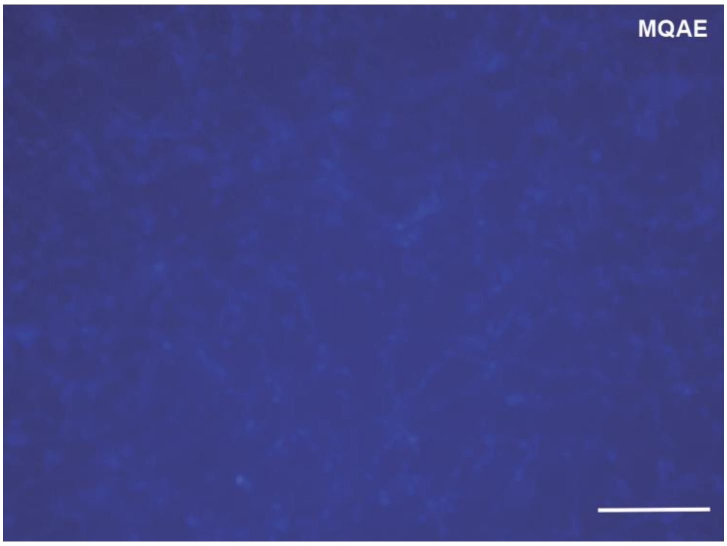

Figure S3. MQAE live stain fail to label hiNSC derived neurons. hiNSC derived day 10 neurons stained with MQAE an intracellular Cl^- ion reporter. Scale bars, 100 μm.

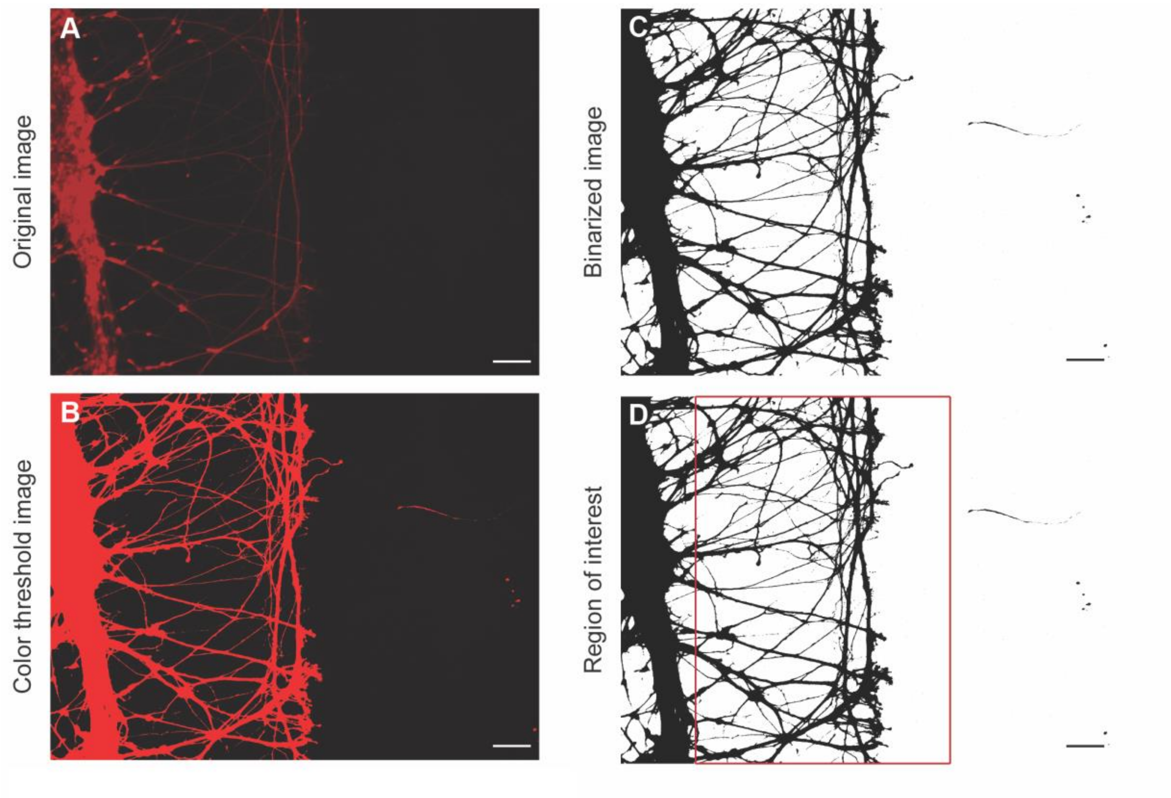

Figure S4. Quantitative determination of neurite outgrowth from scratch injured neurons. hiNSC derived neurons stained with Calcein Red-Orange AM live stain after scratch injury to quantify neurite outgrowth into the scratch. (A) Original Calcein Red-Orange AM image of neurite outgrowth. (B) Same original image after it has undergone color thresholding. (C) Binarization of color thresholded image. (D) Demarcation of region of interest (magenta square) for measurement of integrated density of fluorescent signal in the region of interest. Measure of neurite outgrowth is obtained by dividing integrated density with the area of region of interest. All scale bars, 100 μm.

Table S1. Ionic composition (in mM) of extracellular solutions with changing Na^+ ion concentration and physiological levels in green. Adapted from [85].

NaCl	KCl	$CaCl_2$	$MaCl_2$	NMDG-Cl	HEPES	Glucose
28	5.4	1.8	1	112	10	5.5
56	5.4	1.8	1	84	10	5.5
84	5.4	1.8	1	56	10	5.5
112	5.4	1.8	1	28	10	5.5
140	5.4	1.8	1	0	10	5.5

Na^+	K^+	$NMDG^+$	Cl^-
28	5.4	112	151
56	5.4	84	151
84	5.4	56	151
112	5.4	28	151
140	5.4	0	151

Table S2. Ionic composition (in mM) of extracellular solutions with changing K^+ ion concentration and physiological levels in green. Adapted from [85].

NaCl	KCl	$CaCl_2$	$MaCl_2$	NMDG-Cl	HEPES	Glucose
0	5.4	1.8	1	140	10	5.5
0	35.4	1.8	1	110	10	5.5
0	65.4	1.8	1	80	10	5.5
0	100.4	1.8	1	45	10	5.5
0	135.4	1.8	1	10	10	5.5

Na^+	K^+	$NMDG^+$	Cl^-
0	5.4	140	151
0	35.4	110	151
0	65.4	80	151
0	100.4	45	151
0	135.4	10	151

Table S3. Ionic composition (in mM) of extracellular solutions within changing Na^+ and K^+ ion concentration for generating various resting membrane potentials with physiological levels in green. Adapted from [85].

NaCl	KCl	$CaCl_2$	$MaCl_2$	NMDG-Cl	HEPES	Glucose
140	5.4	1.8	1	0	10	5.5
110	35.4	1.8	1	0	10	5.5
80	65.4	1.8	1	0	10	5.5
45	100.4	1.8	1	0	0	5.5
10	135.4	1.8	1	0	0	5.5

Na^+	K^+	$NMDG^+$	Cl^-
140	5.4	0	151
110	35.4	0	151
80	65.4	0	151
45	100.4	0	151
10	135.4	0	151

References

85. Bonzanni, M.; Payne, S.L.; Adelfio, M.; Kaplan, D.L.; Levin, M.; Oudin, M.J. Defined extracellular ionic solutions to study and manipulate the cellular resting membrane potential. *Biol. Open* **2020**, *9*, 1.
